# Influence of body mass index on the innate immune response during human endotoxemia

**DOI:** 10.1186/cc9667

**Published:** 2011-03-11

**Authors:** R Van der Pluijm

**Affiliations:** 1 UMC St Radboud, Nijmegen, the Netherlands

## Introduction

Accumulating data suggest a protective effect of obesity in the case of severe infections. Higher baseline levels of the proinflammatory cytokine TNFα as well as more pronounced TNFα release following whole blood stimulation with endotoxin are reported in patients with a higher body mass index (BMI). This more pronounced proinflammatory response in obese patients may enable a rapid and more effective clearance of microbial pathogens. The effect of the body mass index on the innate immune response *in vivo *has not been assessed.

## Methods

The immune response and BMI of 69 healthy subjects that were included in several experimental endotoxemia studies were analyzed. Endotoxemia was induced by the administration of 2 ng/kg *Escherichia coli *lipopolysaccharide. Concentrations of TNFα and IL-10 were serially determined (Luminex assay). Areas under the curve of cytokine levels were calculated and analyzed with unpaired *t *tests. All data are expressed as mean ± SEM of *n *subjects.

## Results

**Figure 1:**
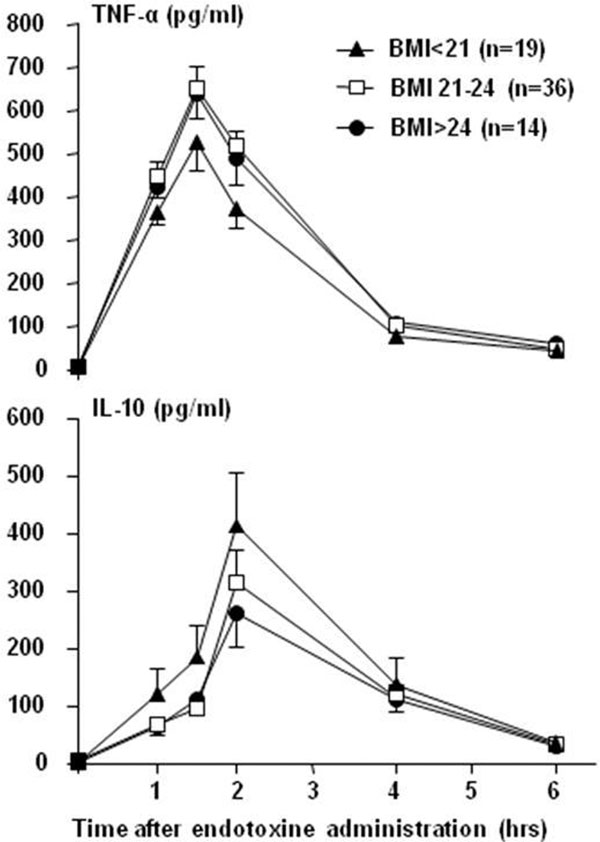
**Effects of 2 ng/kg *Escherichia coli *endotoxin (LPS) (administered at 0 hours) in subjects with BMI <21 and BMI >24 kg/m^2 ^on the production of TNFα and IL-10**. Data expressed as mean ± SEM.

**Figure 2:**
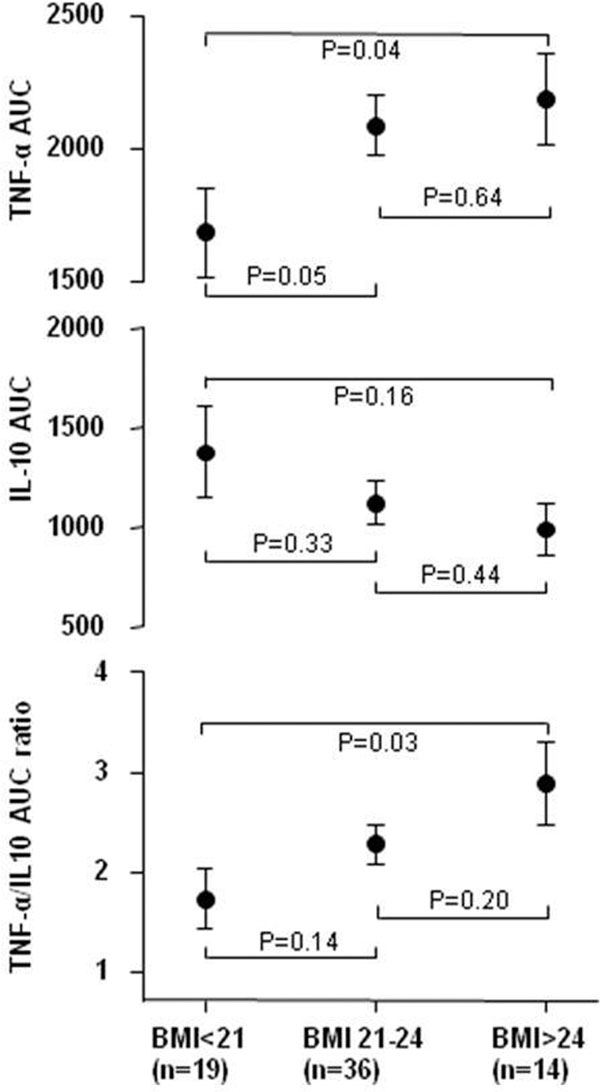
**AUC of TNFα and IL-10 and the TNFα/IL-10 ratio in subjects with BMI <21, BMI 21 to 24 and BMI >24 kg/m^2^**. Data expressed as mean ± SEM.

All subjects showed increased production of both proinflam-matory cytokine TNFα and anti-inflammatory cytokine IL-10 (Figure 1). The area under the curve of TNFα levels was related to the BMI (Figure 2) as subjects with BMI >24 kg/m^2 ^released more TNFα than those with BMI <21 kg/m^2 ^(*P *= 0.04). An opposite trend of IL-10 levels was observed in association with higher BMI (*P *= 0.12). The quotient of TNFα/IL-10 AUC levels, serving as a readout of the pro/anti-inflammatory balance of a subject, showed a more proinflammatory response in subjects with a higher BMI compared with those with a lower BMI (*P *= 0.03) (Figure 2).

## Conclusions

This study is the first to demonstrate that a higher BMI is associated with a shift in the pro/anti-inflammatory balance towards a more pronounced proinflammatory immune response in humans *in vivo*.

